# Effect of *GBA* Mutations on Phenotype of Parkinson's Disease: A Study on Chinese Population and a *Meta-Analysis*

**DOI:** 10.1155/2015/916971

**Published:** 2015-09-02

**Authors:** Yuan Zhang, Qi-ying Sun, Yu-wen Zhao, Li Shu, Ji-feng Guo, Qian Xu, Xin-xiang Yan, Bei-sha Tang

**Affiliations:** ^1^Department of Neurology, Xiangya Hospital, Central South University, Changsha, Hunan 410008, China; ^2^Department of Geriatrics, Xiangya Hospital, Central South University, Changsha, Hunan 410008, China; ^3^Key Laboratory of Hunan Province in Neurodegenerative Disorders, Changsha, Hunan 410008, China; ^4^State Key Laboratory of Medical Genetics, Changsha, Hunan 410008, China; ^5^Parkinson's Disease Center, Beijing Institute for Brain Disorders, Capital University of Medical Sciences, Beijing 100069, China

## Abstract

GBA has been identified as a genetic risk factor for PD. Whether the clinical manifestations of PD patients with or without GBA mutations are different has still not reached a consensus. We firstly detected the GBA mutation L444P in 1147 Chinese PD patients and simultaneously evaluated their corresponding clinical data. Then we compared the phenotypes between 646 PD patients with GBA mutations and 10344 PD patients without GBA mutations worldwide through *meta-analysis*. Through the method of *meta-analysis*, there was significant difference in age at onset (MD = −3.10 [95% CI: −4.88, −1.32]), bradykinesia as an initial symptom (OR = 1.49 [95% CI: 1.15, 1.94]), having family history (OR = 1.50 [95% CI: 1.18, 1.91]), and dementia (OR = 3.21 [95% CI: 1.97, 5.24]) during the comparison between PD patients with and without GBA mutations. While, in the aspect of tremor as an initial symptom (OR = 0.81 [95% CI: 0.64, 1.03]), the severity of motor symptoms such as H-Y (MD = 0.06 [95% CI: −0.06, 0.17]) and UPDRS-III (MD = 1.61 [95% CI: −0.65, 3.87]) and having dyskinesia (OR = 1.60 [95% CI: 0.90, 2.84]) during the comparison between the two groups revealed no statistical differences. Our results suggested that the phenotypes of PD patients with GBA mutations are different from GBA noncarriers.

## 1. Introduction

Parkinson's disease (PD) is the second most common progressive neurodegenerative disorder. Though the etiology of PD remains unclear, there is an increasing evidence that genetic-factor contributes to the etiology of PD.

Mutations in the gene encoding the lysosomal enzyme glucocerebrosidase (*GBA*) have been identified as a genetic risk factor for PD [1]. Aharon-Peretz and her colleagues [2] reported that the overall clinical manifestations and age at disease onset have no differences in PD patients with* GBA* mutations (*GBA *+ PD) compared with those without mutations (*GBA* − PD) in 148 Ashkenazi PD patients. However, recent researches have shown that the clinical features of* GBA *+ PD differ from* GBA* − PD to some extent. Winder-Rhodes et al. [3] showed that* GBA* carriers in PD were inclined to suffer an earlier age at onset (AAO) and more severe nonmotor and motor symptoms. Hu and other researchers [4] reported that* GBA* mutations influenced the course of PD with respect to the appearance of dementia. Whether the clinical manifestations of PD patients with or without* GBA* mutations are different or not has still not reached a consensus.

In order to evaluate the effect of* GBA* on the phenotype of PD, we firstly explored the relationship between* GBA* mutations and their clinical characteristics in Chinese PD patients. Then the method of* meta-analysis* was used to assess the possible role of* GBA* in the phenotype of PD with larger sample size worldwide.

## 2. Methods

### 2.1. Subjects and Clinical Characteristics

1147 PD patients were collected continuously from 2005 to 2014 from the outpatient neurology clinics of Xiangya Hospital of China. PD was diagnosed by two or more experienced neurologists according to the United Kingdom Parkinson Disease Society Brain Bank criteria (UKBB) [5] with the exception that a positive family history was not a part of the exclusion criteria. None had a history of neurologic or psychiatric conditions other than PD. We collected age at onset and initial motor symptom from all participants. Other assessments were blindly performed during the “on” motor state. Hoehn-Yahr rating scale (H-Y) and part III of unified Parkinson disease rating scale (UPDRS-III) were used to evaluate the severity of 424 PD patients' motor symptoms, who were continuously collected from 2011 to 2014. The presentation of dyskinesia was evaluated through UPDRS-IV in 424 PD patients, continuously collected from 2011 to 2014. Dementia status of 268 PD patients who were continuously collected from 2013 to 2014 was evaluated according to the Movement Disorder Society task force (MDS-TF) consensus criteria. All clinical information was shown in Table 1.

### 2.2. Genetic Analysis

All 1147 PD patients were detected for* GBA* gene L444P mutation, the most common PD-associated* GBA* mutation in Chinese population [24]. The screening procedures and details have been reported recently [25].

### 2.3. Meta-Analysis

Including our own data of Chinese population, a* meta-analysis* related to the above topic was conducted. Eligible studies had to meet the following criteria: (1) being a case-control study except for reviews, case reports, editorials, or functional researches; (2) all PD patients being diagnosed according to UKBB criteria with the exception that a positive family history was not a part of the exclusion criteria; (3) only including publications related to* GBA* mutation analysis; (4) clearly reporting results of* GBA* mutations and corresponding clinical data. Then we searched electronic databases including Embase, PubMed, Cochrane Library, and Web of Knowledge and Wanfang database and CNKI up to May 1, 2015, using combination of following keywords:* GBA*, glucocerebrosidase, and Parkinso^*∗*^ both in English and in Chinese. Reference lists and personal communications of authors were also referred to as sources to include articles cited elsewhere.

**Figure 1:**
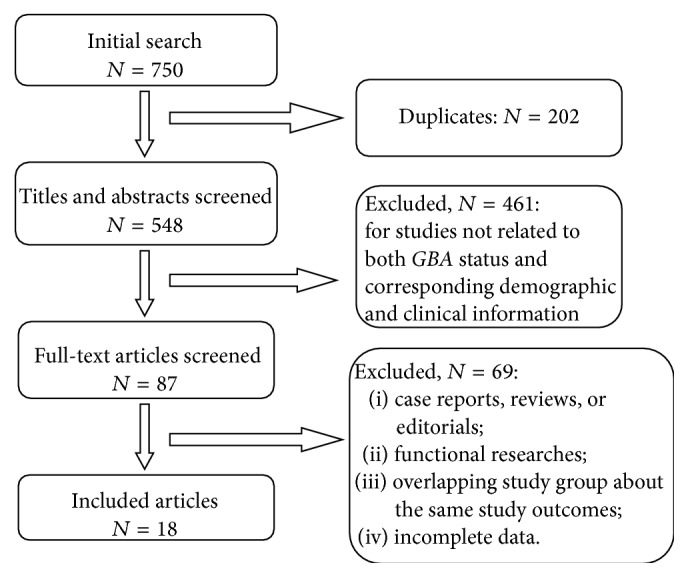
Flowchart of included publications.

To select studies for further assessment, two authors independently scanned the abstracts, titles, or both sections of each retrieved record. All potentially relevant articles were investigated in full text. For studies satisfying the aforesaid criteria, two authors independently abstracted the following data: year of publication, first author's surname, country of participants, numbers of PD patients with and without* GBA* mutations, and corresponding clinical information. The flowchart of studies selection and reasons for exclusion are presented in Figure 1. The qualities of the included studies were evaluated by the Newcastle-Ottawa Scale (NOS) [26].

In order to assess the strength of association between* GBA* mutations and clinical manifestation, dichotomous outcome was expressed as odds ratio (OR) while continuous outcome was expressed as mean difference (MD) with 95% confidence intervals (CI). Heterogeneity across individual studies was identified by a standard *Q* test with a significance level of *α* = 0.1 and *I* ^2^. If heterogeneity did not exist (*Q* > 0.10) or the severity of heterogeneity was accepted (*I* ^2^ ≤ 50%), the fixed effect model was adopted to calculate the pooled OR and MD value. Otherwise, the random effect model was used. Funnel-plot analysis was used to assess the reporting bias. All analyses were carried out by using the Review Manager software package v.5.3 (The Cochrane Collaboration, Oxford, England).

## 3. Results

34* GBA *+ PD and 1113* GBA* − PD were available in our own data from Chinese. A total of 18 eligible studies were included during the process of* meta-analysis.* The main characteristics of all included studies are summarized in Table 1.

### 3.1. Onset Characteristics

#### 3.1.1. Family History

**Figure 2:**
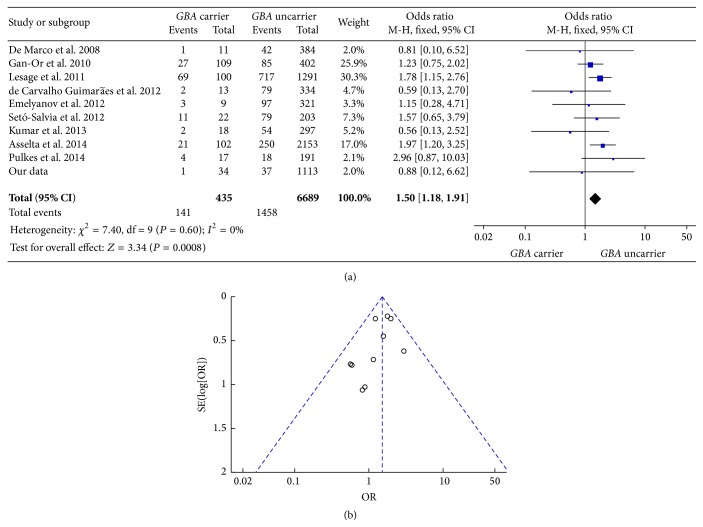
(a) Forest plot of family history in* GBA* + PD and* GBA* − PD. (b) Funnel plot of family history in* GBA* + PD and* GBA* − PD.

PD patients with family history are defined as having at least one first- or second-degree relative with the diagnosis of PD. There are 38 PD patients (1* GBA *+ PD, 37* GBA* − PD) with family history in our own data. We included another 9 publications to assess the relationship between* GBA* status and family history of PD patients. The pooled OR of family history in* GBA *+ PD and* GBA* − PD was 1.50 [95% CI: 1.18, 1.91] (Figure 2(a)). That means* GBA *+ PD was more likely to be exposed to family history than* GBA* − PD, even though the relationship was negative in our own data. The funnel plot was symmetric (Figure 2(b)).

#### 3.1.2. Age at Onset (AAO)

**Figure 3:**
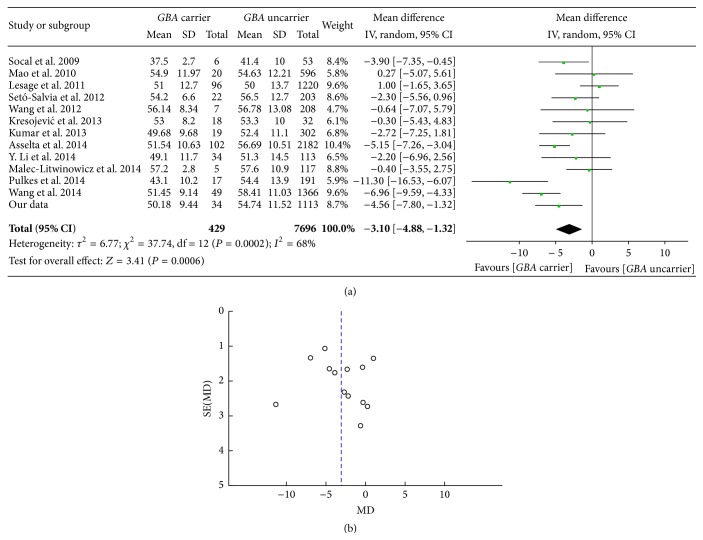
(a) Forest plot of age at onset in* GBA* + PD and* GBA* − PD. (b) Funnel plot of age at onset in* GBA* + PD and* GBA* − PD.

A total of 429* GBA* carriers and 7696* GBA* uncarriers were included in the analysis of relationship between AAO of PD patients and* GBA* status. The pooled MD of AAO between* GBA *+ PD and* GBA* − PD was −3.10 [95% CI: −4.88, −1.32] (Figure 3(a)). It means that the AAO is nearly 3 years earlier in* GBA *+ PD than* GBA* − PD. The funnel plot was asymmetric and the bias leaned to no difference in the two groups (Figure 3(b)). The AAO of* GBA *+ PD and* GBA* − PD are 50.18 ± 9.44 and 54.74 ± 11.52 individually in our own data from Chinese population, which was consistent with the results of* meta-analysis.*

#### 3.1.3. Initial Motor Symptom

**Figure 4:**
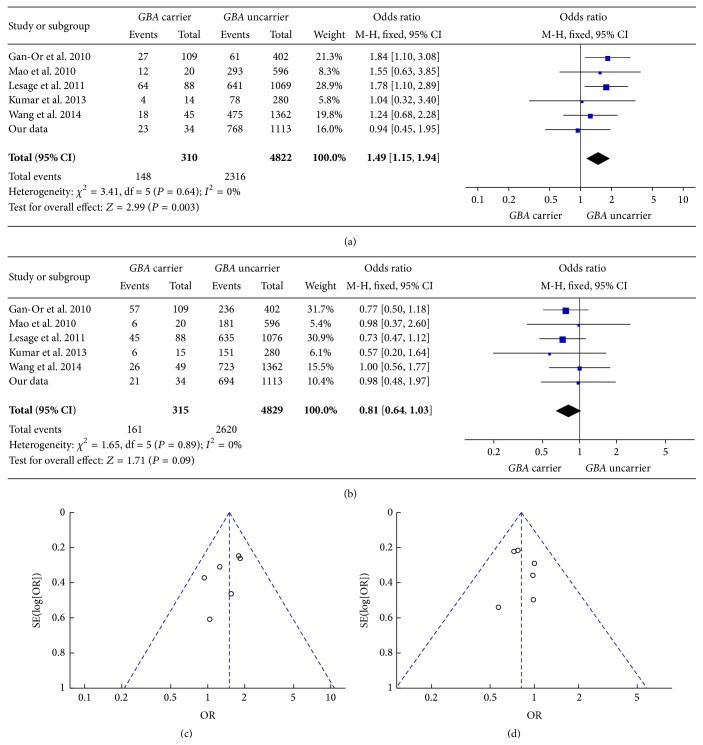
(a) Forest plot of bradykinesia as an initial symptom in* GBA* + PD and* GBA* − PD. (b) Forest plot of tremor as an initial symptom in* GBA *+ PD and* GBA* − PD. (c) Funnel plot of bradykinesia as an initial symptom in* GBA *+ PD and* GBA* − PD. (d) Funnel plot of tremor as an initial symptom in* GBA *+ PD and* GBA* − PD.

We analyzed two common initial motor symptoms: bradykinesia and tremor. The pooled OR of bradykinesia as an initial symptom between* GBA *+ PD and* GBA* − PD was 1.49 [95% CI: 1.15, 1.94] (Figure 4(a)). The pooled OR of tremor as an initial symptom between* GBA *+ PD and* GBA* − PD was 0.81 [95% CI: 0.64, 1.03] (Figure 4(b)). The two funnel plots were symmetric (Figures 4(c) and 4(d)). Unfortunately, there was no significant difference in our own data from Chinese population, which was consistent with publications that referred to Chinese population [6, 20].

### 3.2. Progression Features

#### 3.2.1. Severity of PD Motor Symptoms

**Figure 5:**
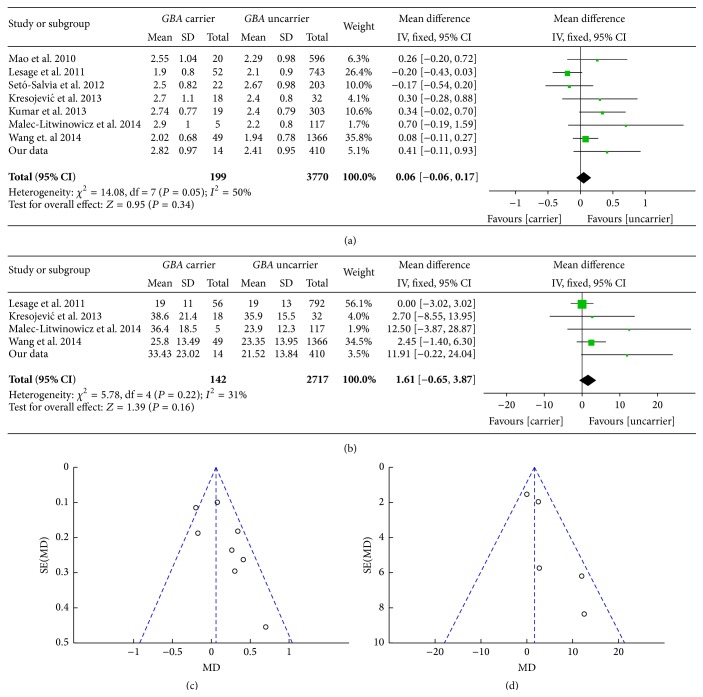
(a) Forest plot of H-Y in* GBA *+ PD and* GBA* − PD. (b) Forest plot of UPDRS-III in* GBA *+ PD and* GBA* − PD. (c) Funnel plot of H-Y in* GBA *+ PD and* GBA* − PD. (d) Funnel plot of UPDRS-III in* GBA *+ PD and* GBA* − PD.

We continuously evaluated the H-Y and UPDRS-III in 14* GBA *+ PD and 410* GBA* − PD in Chinese mainland population from 2011 to 2014. Together with the included publications' data, the pooled MD of H-Y between* GBA *+ PD and* GBA* − PD was 0.06 [95% CI: −0.06, 0.17] (Figure 5(a)). The pooled MD of UPDRS-III among the two groups was 1.61 [95% CI: −0.65, 3.87] (Figure 5(b)). The two funnel plots were asymmetric which tended to positive results (Figures 5(c) and 5(d)). The MDs of H-Y and UPDRS-III showed no significant differences between* GBA* carriers and uncarriers in mainland Chinese population and other centers.

#### 3.2.2. Dementia

**Figure 6:**
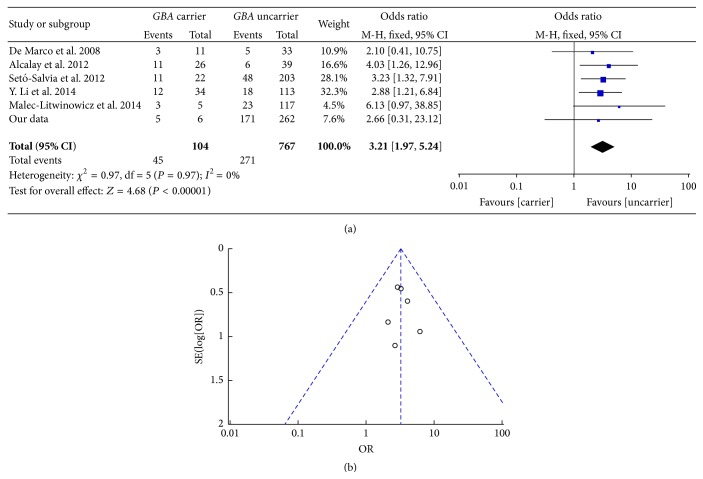
(a) Forest plot of dementia in* GBA *+ PD and* GBA* − PD. (b) Funnel plot of dementia in* GBA *+ PD and* GBA* − PD.

We found 176 PD patients (5* GBA *+ PD and 171* GBA* − PD) with dementia among the 268 PD patients (6* GBA *+ PD and 262* GBA* − PD), continuously collected from 2013 to 2014, whose dementia status was evaluated through the Movement Disorder Society task force (MDS-TF) consensus criteria. Dementia status of participants was evaluated through Clinical Dementia Rating (CDR) scale [14, 17] and MMSE [8, 9] or reported by author [27] in included publication. Even though all the dementia statuses were defined by various rating scales, the heterogeneity of all included publications was accepted when we made the* meta-analysis*. The pooled OR of dementia between* GBA *+ PD and* GBA* − PD was 3.21 [95% CI: 1.97, 5.24] (Figure 6(a)) and the funnel plot was symmetric (Figure 6(b)). The result means that PD patients with* GBA* mutations are 3 times more likely than uncarriers to develop dementia.

#### 3.2.3. Dyskinesia

**Figure 7:**
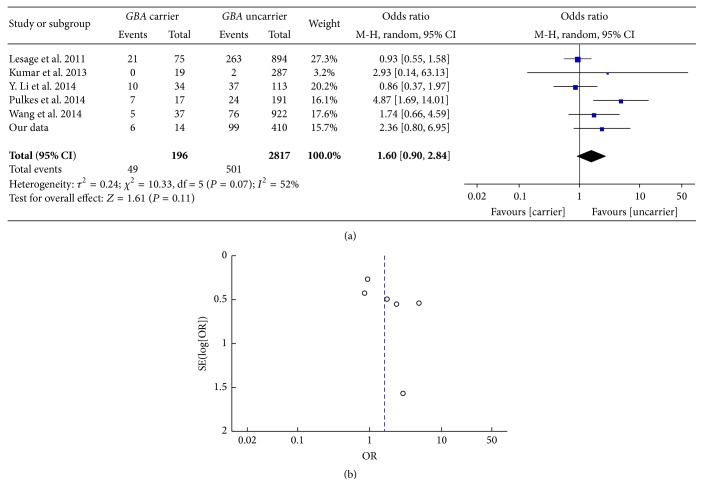
(a) Forest plot of dyskinesia in* GBA *+ PD and* GBA* − PD. (b) Funnel plot of dyskinesia in* GBA *+ PD and* GBA* − PD.

We evaluated the presentation of dyskinesia among 424 PD patients (14* GBA *+ PD and 410* GBA* − PD) through UPDRS-IV, who were continuously collected from 2011 to 2014. 105 PD patients (6* GBA *+ PD and 99* GBA* − PD) presented with dyskinesia. Besides, 5 publications were included to illustrate the connection between dyskinesia and* GBA* status in PD. The pooled OR of dyskinesia between* GBA *+ PD and* GBA* − PD was 1.64 [95% CI: 0.91, 2.94] (Figure 7(a)). The funnel plot was symmetric (Figure 7(b)).

## 4. Discussion

We firstly presented an extensive and detailed phenotype description of* GBA *+ PD in Chinese together with* meta-analysis* worldwide, focusing on not only the disease onset features but also disease progression characteristics. The phenotype of* GBA *+ PD, sharing a spectrum of Parkinsonian phenotype, differs from* GBA* − PD in the following aspects: AAO, initial motor symptom, presentation with family history, and dementia.

Totally, 646* GBA *+ PD and 10344* GBA* − PD were included. The NOS scores of all included publications were rated from 6 stars to 9 stars, which showed that none of the included publications were of low quality.

Additionally, there was no obvious publication reporting bias from the symmetric funnel plots related to the analysis in family history, bradykinesia or tremor as an initial symptom, dementia, and dyskinesia. Even though there was no different in the funnel plot of AAO, we still figured out the earlier onset age of* GBA *+ PD than that of* GBA* − PD. Thus, we can conclude that the* GBA *+ PD patients are more likely to onset with earlier age than* GBA *− PD, which are easily ignored by clinical doctors because PD is more likely to develop in the old. In the same way, the MDs of H-Y and UPDRS –III showed no significant differences with so many positive reports. That is to say,* GBA* mutations in PD patients are not related to the severity in PD.

What is more, both genetic and clinical heterogeneities exist during the analysis process. All heterogeneities may originate from the following parts: firstly, the frequency of the different* GBA* mutations varies according to ethnicity [27]. For instance, in Ashkenazi Jewish (AJ), N370S is the most frequent mutation, whereas the L444P is more common in Asian population than others. Still, other variations, like R120W and D409H, were also reported in several studies but rarely showed positive outcomes. We could not extract single* GBA* mutation and corresponding clinical information from included publications. Thus, mixed* GBA* carriers and their clinical data together may affect the final results, especially for the ethnicity related data. Even in the same ethnicity, the heterogeneity also could not be avoided due to different genetic screening methods, other susceptible genes apart from* GBA*, and different degree of effects by the different* GBA* mutations [28]. Secondly, studies which did not adjust for age, sex, disease duration, environmental exposures, and other cofactors may influence clinical phenotype of PD.

The foundation underlying the relation of* GBA* genotype to clinical characteristics of PD remains elusive. Mutant alleles of* GBA* can result in widespread deficiency of the enzymatic activity that might be involved in abnormal synuclein aggregation [1]. Besides, both histopathologic and positron emission tomography studies have suggested that* GBA* carriers were significantly more likely than noncarriers to have diffused Lewy bodies which might be associated with a higher prevalence and severity of cognitive impairment and neuropsychiatric characteristics [29, 30].

## 5. Conclusion

In conclusion, our results suggest that PD phenotype of* GBA* mutation carriers is more likely to appear with family history, earlier AAO, bradykinesia as an initial symptom, and presentation with dementia compared with those uncarriers. Further studies, especially cofactors, matched single mutation of* GBA*, and its correspondent clinical data are needed to further illustrate the relationship.

## Figures and Tables

**Table 1:** Results of our own data in Chinese and attributes of all included studies.

First author	Country	Group	Number	Family history	AAO (*n*)^a^	Tremor (*n*)^a^	Bradykinesia (*n*)^a^	H-Y (*n*)^a^	UPDRS-III (*n*)^a^	Dementia (*n*)^a^	Dyskinesia (*n*)^a^
Our own data	China	*GBA* + PD	34	1	50.18 ± 9.44	21	23	2.82 ± 0.97 (14)	33.43 ± 23.02 (14)	5 (6)	6 (14)
		*GBA* − PD	1113	37	54.75 ± 11.52	694	768	2.41 ± 0.95 (410)	21.55 ± 3.84 (410)	171 (262)	99 (410)

Wang 2014 [6]	China	*GBA* + PD	49	—	51.45 ± 9.14	26 (49)	18 (45)	2.02 ± 0.68	25.80 ± 13.49	—	5 (37)
		*GBA* − PD	1366	—	58.41 ± 11.03	723 (1362)	475 (1362)	1.94 ± 0.78	23.35 ± 13.95	—	76 (922)

Pulkes 2014 [7]	China	*GBA* + PD	17	4	43.1 ± 10.2	—	—	—	—	—	7
		*GBA* − PD	191	18	54.4 ± 13.9	—	—	—	—	—	24

Malec-Litwinowicz 2014 [8]	Poland	*GBA* + PD	5	—	57.2 ± 2.8	—	—	2.9 ± 1.0	36.4 ± 18.5	3	—
		*GBA* − PD	171	—	57.6 ± 10.9	—	—	2.2 ± 0.8	23.9 ± 12.3	23	—

Li 2014 [9]	Japan	*GBA* + PD	34	34	49.1 ± 11.7	—	—	—	—	12	10
		*GBA* − PD	113	113	51.3 ± 14.5	—	—	—	—	18	37

Asselta 2014 [10]	Italy	*GBA* + PD	102	21	51.54 ± 10.63	—	—	—	—	—	—
		*GBA* − PD	2182	250 (2153)	56.69 ± 10.51	—	—	—	—	—	—

Kumar 2013 [11]	Serbia	*GBA* + PD	21	2 (18)	49.68 ± 9.68 (19)	6 (15)	4 (14)	2.74 ± 0.77 (19)	—	—	0 (19)
		*GBA* − PD	339	54 (297)	52.40 ± 11.1 (302)	151 (280)	78 (280)	2.40 ± 0.79 (303)	—	—	2 (287)

Kresojević 2013 [12]	Serbia	*GBA* + PD	18	—	53.0 ± 8.2	—	—	2.7 ± 1.1	38.6 ± 21.4	—	—
		*GBA* − PD	32	—	53.3 ± 10.0	—	—	2.4 ± 0.8	35.9 ± 15.5	—	—

Wang 2012 [13]	China	*GBA* + PD	7		56.14 ± 8.34	—	—	—	—	—	—
		*GBA* − PD	208		56.78 ± 13.08	—	—	—	—	—	—

Setó-Salvia 2012 [14]	Europe	*GBA* + PD	22	11	54.2 ± 6.6	—	—	2.5 ± 0.82	—	11	—
		*GBA* − PD	203	79	56.5 ± 12.7	—	—	2.67 ± 0.98	—	48	—

Emelyanov 2012 [15]	Russia	*GBA* + PD	9	3	—	—	—	—	—	—	—
		*GBA* − PD	321	97	—	—	—	—	—	—	—

de Carvalho Guimarães 2012 [16]	Brazil	*GBA* + PD	13	2	—	—	—	—	—	—	—
		*GBA* − PD	334	79	—	—	—	—	—	—	—

Alcalay 2012 [17]	Multisites	*GBA* + PD	33	—	—	—	—	—	—	11 (26)	—
		*GBA* − PD	114	—	—	—	—	—	—	6 (39)	—

Lesage 2011 [18]	Europe	*GBA* + PD	100	69	51.0 ± 12.7 (96)	45 (88)	64 (88)	1.9 ± 0.8 (52)	19 ± 11 (56)	—	21 (75)
		*GBA* − PD	1291	717	50.0 ± 13.7 (1220)	635 (1076)	641 (1069)	2.1 ± 0.9 (743)	19 ± 13 (792)	—	263 (894)

Huang 2011 [19]	China	*GBA* + PD	36	—	—	—	—	—	—	—	—
		*GBA* − PD	931	—	—	—	—	—	—	—	—

Mao 2010 [20]	China	*GBA* + PD	20	—	54.90 ± 11.97	6 (20)	12 (20)	2.55 ± 1.04	—	—	—
		*GBA* − PD	596	—	54.63 ± 12.21	181 (596)	293 (596)	2.29 ± 0.98	—	—	—

Gan-Or 2010 [21]	Israel	*GBA* + PD	109	27	—	57	27	—	—	—	—
		*GBA* − PD	402	85	—	236	61	—	—	—	—

Socal 2009 [22]	Brazil	*GBA* + PD	6	—	37.5 ± 2.7	—	—	—	—	—	—
		*GBA* − PD	53	—	41.4 ± 10	—	—	—	—	—	—

De Marco 2008 [23]	Italy	*GBA* + PD	11	—	—	—	—	—	—	3	—
		*GBA* − PD	384	—	—	—	—	—	—	5	—

(*n*)^a^: number of patients whose clinical information was available.
